# Unveiling the Multifaceted Mechanisms of Antibacterial Activity of Buforin II and Frenatin 2.3S Peptides from Skin Micro-Organs of the Orinoco Lime Treefrog (*Sphaenorhynchus lacteus*)

**DOI:** 10.3390/ijms19082170

**Published:** 2018-07-25

**Authors:** Carolina Muñoz-Camargo, Vivian A. Salazar, Laura Barrero-Guevara, Sandra Camargo, Angela Mosquera, Helena Groot, Ester Boix

**Affiliations:** 1 Department of Biomedical Engineering, Universidad de los Andes, Bogotá 111711, Colombia; c.munoz2016@uniandes.edu.co; 2 Laboratorio de Genética Humana, Department of Biological Sciences, Universidad de los Andes, Bogotá 111711, Colombia; visalazar@uniandes.edu.co (V.A.S.); la.barrero1854@uniandes.edu.co (L.B.-G.); sandracamargouis@gmail.com (S.C.); 3 Department of Biochemistry and Molecular Biology, Faculty of Biosciences, Universitat Autònoma de Barcelona, 08193 Cerdanyola del Vallès, Spain; angela.mosquera@udea.edu.co; 4 Institute of Life Sciences, Hebrew University of Jerusalem, Jerusalem 91904, Israel; 5 Biotechnology Group, Biology Institute, Universidad de Antioquia, Medellín 050010, Colombia

**Keywords:** antibacterial activity, antimicrobial peptides, bacterial agglutination, buforin II, DNA binding, frenatin 2.3S, frog skin secretions, membrane translocation, membrane leakage

## Abstract

Amphibian skin is a rich source of natural compounds with diverse antimicrobial and immune defense properties. Our previous studies showed that the frog skin secretions obtained by skin micro-organs from various species of Colombian anurans have antimicrobial activities against bacteria and viruses. We purified for the first time two antimicrobial peptides from the skin micro-organs of the Orinoco lime treefrog (*Sphaenorhynchus lacteus*) that correspond to Buforin II (BF2) and Frenatin 2.3S (F2.3S). Here, we have synthesized the two peptides and tested them against Gram-negative and Gram-positive bacteria, observing an effective bactericidal activity at micromolar concentrations. Evaluation of BF2 and F2.3S membrane destabilization activity on bacterial cell cultures and synthetic lipid bilayers reveals a distinct membrane interaction mechanism. BF2 agglutinates *E. coli* cells and synthetic vesicles, whereas F2.3S shows a high depolarization and membrane destabilization activities. Interestingly, we found that F2.3S is able to internalize within bacterial cells and can bind nucleic acids, as previously reported for BF2. Moreover, bacterial exposure to both peptides alters the expression profile of genes related to stress and resistance response. Overall, these results show the multifaceted mechanism of action of both antimicrobial peptides that can provide alternative tools in the fight against bacterial resistance.

## 1. Introduction

The alarming increase in the frequency of bacterial resistance to conventional antibiotics during the last decade is nowadays one of the main global health concerns. The search for novel antimicrobial drugs is a top priority for the World Health Organization (WHO). In this context, the host defense peptides (HDP) or antimicrobial peptides (AMPs) have attracted particular interest from the pharmaceutical industry. AMPs are predominantly cationic and contain a high percentage of hydrophobic residues. These features confer structural characteristics to the peptides that define their biological activity and mechanisms of action, as well as represent a basis of new useful natural antibiotics for pharmaceutical applications [1,2,3]. Overall, two main general mechanisms have been described for accomplishing their antimicrobial activity. In brief, the first is involved in bacterial membrane disruption and the second targets intracellular components such as nucleic acids following the peptide internalization [4]. 

**Figure 1:**
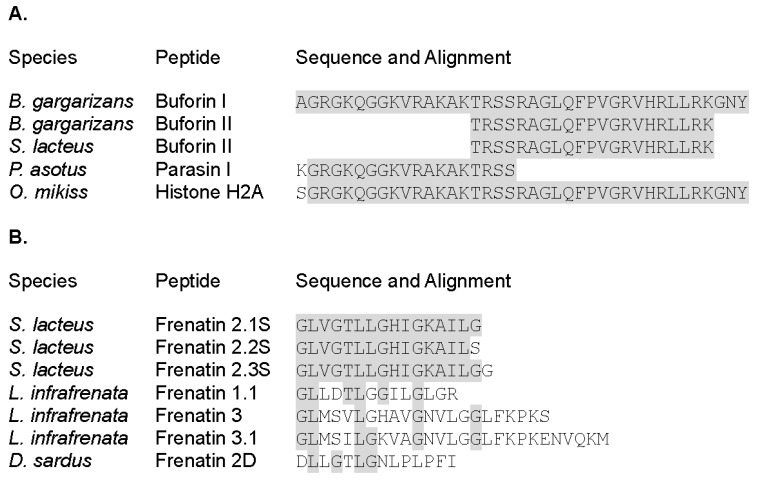
Alignment of peptides BF2 and F2.3S purified from *S. lacteus* and their homologues. (**A**) Primary sequences of buforins I and II from *S. lacteus*, *Bufo bufo gargarizans* and homologues found in the fishes *Parasilurus asotus* and *Oncorhynchus mikiss*; (**B**) Comparison of primary structures of F2.3S with their amphibian homologs, frenatins from *Litoria* genus and *Discoglossus sardus*. *Clustal O* (1.2.4) multiple sequence alignment. Identities are indicated in grey.

**Figure 2:**
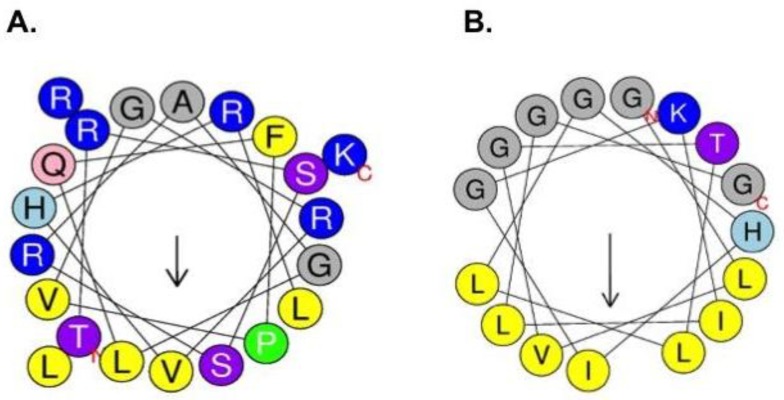
Helical wheel projections of BF2 (**A**) and F2.3S (**B**). Cationic residues are highlighted in blue, uncharged residues in grey, polar residues in purple, nonpolar residues in yellow, polar/uncharged residues in pink and anionic residues in red. N-terminal and C-terminal domains are indicated with N and C letters respectively. The directions of hydrophobic moments of the peptides are denoted by the arrows in the middle of the wheels. Drawn by *HeliQuest* (available online: http://heliquest.ipmc.cnrs.fr/). BF2 secondary structure comprises a distorted-helix between residues 7–11 and a regular α-helix between residues 12–20 [7] and F2.3S secondary structure encompasses a unique α-helix between residues 3–15 (see the secondary structure prediction in Figure S1).

AMPs are often truncated versions of larger proteins within the cell. For example, histones are proteins known for their function in the cell nucleus; nevertheless, they also can be found in the cytoplasm, and the fragments of these proteins have been identified in frog skin secretions. The proteolytic fragments were named histone-derived peptides and reported to display antimicrobial and inflammatory properties [5]. One of the most studied histone derived peptides families are buforins (Figure 1). Buforins are released by histone H2A proteolytic cleavage, together with other peptides, such as parasins and hipposins. Within the buforin family the buforin II peptide (BF2), derived from buforin I cleavage, displays a prominent antimicrobial activity and has attracted particular interest. BF2 was first isolated from the stomach secretion of *Bufo bufo gargarizans* [6]; the AMP has a primary sequence of 21 amino acids that adopts a helix-hinge-helix structure in a membrane mimic environment (Figure 2) [7]. BF2 antibacterial activity has been reported against both Gram-positive and Gram-negative bacteria. BF2 kills bacteria by entering the cells in a non-lytic manner and binding to nucleic acids [8]. Interestingly, BF2 membrane translocation is independent of any cellular receptor and the peptide can readily enter both bacterial cells and liposomes [9,10]. Previous studies have shown the important role of proline in the ability of the peptide to translocate across the bacterial membrane by the formation of transient toroidal pores [11,12,13,14]. Furthermore, some studies have shown that buforin can act synergistically with other antibiotics such as cefazolin, thereby increasing the antibacterial action and its activity against biofilms [12,15].

On the other hand, the AMP frenatin family was first described in the skin secretions from the Australian frog *Litoria infrafrenata* [16]. Four frenatin peptides were identified as antimicrobial active peptides with an unrelated primary structure to previous reported amphibian AMPs [16]. The novel treefrog peptides attracted the researcher interest because of their similarity with mammalian peptides. Interestingly, later studies reported immunomodulatory and antitumoral properties for frenatins [17,18]. Three frenatin sequences have been identified in *Sphaenorhynchus lacteus* (Figure 1). The peptides are 16 to 17 amino acid long and share a high sequence identity, showing punctual variations at the C-terminus. In particular, frenatin F2.3S was identified in our laboratory from in vitro skin micro-organs (SMOs) of *S. lacteus* and shares low sequence identity with previously described frenatins 1 and 3 [19,20], such as frenatin 1 from *L. infrafrenata* and Frenatin 2D from *Discoglossus sardus* [16,21]. In contrast, frenatin 2.1S, 2.2S and 2.3S from *S. lacteus* conform a highly homologous group with reported antimicrobial activity against both Gram-positive and Gram-negative bacteria, including reference species and clinical strains [16,22]. Moreover, our group found that F2.3S has the capacity to protect cells from yellow fever virus infection [19]; a property that might be related to the immunomodulatory properties reported for the peptides of frenatin family [18]. The immunomodulatory activity was reported especially for frenatin 2.1 due to its capacity to differentially stimulate macrophages in order to produce pro-inflammatory (TNFα, IL-23, IL-6) and anti-inflammatory (IL-10) cytokines in mononuclear cells of mouse peritoneum and spleen [23]. In spite of the previous studies about the antimicrobial and immunomodulatory activities of frenatins [19], the mechanism of action of F2.3S remains unknown. 

Frogs and toads have developed under evolutionary selective pressure a successful strategy for surviving in microbe-laden hostile environments, which relies heavily on the secretion of chemical cocktails of AMPs from specialized granular skin glands [24]. Therefore, the skin secretions of amphibians work as a biological barrier against infection and are an extensive source of promissory AMPs. Several studies have described hundreds of these peptides and their biological activities, including antimicrobial activity, immunomodulation, chemotaxis, and cytotoxicity. Our research group has focused on the search of AMPs among the compounds secreted by common species of anurans widely distributed in Colombia. In this context, the Orinoco lime treefrog (*S. lacteus*), belonging to the Hylidae family, was considered as an attractive specie for our studies. The AMPs secreted by *S. lacteus* frog SMOs were identified and characterized. Within the purified mixture two highly active peptides caught our attention: BF2, showing the same primary sequence that the BF2 isolated from *Bufo bufo gargarizans* stomach tissue, and a peptide belonging to the frenatin family, named frenatin 2.3S (F2.3S).

The present study describes the characterization of BF2 and F2.3S peptides identified in SMOs of *S. lacteus* and analyzes their antimicrobial activity against Gram-positive and Gram-negative species, their action on membrane models and their potential intracellular targets. Our results highlight that the two peptides are multifaceted molecules that can cover broad strategies to act against bacterial pathogens. Both peptides show a combined membrane destabilization and intracellular mechanism of action. Our results support the use of frog skin secretions as a rich source of multifaceted peptides to develop alternative antimicrobial agents.

## 2. Results

### 2.1. Synthesis and Characterization of Physicochemical Properties of BF2 and F2.3S Peptides

In this study we have identified and evaluated BF2 and F2.3S peptides secreted by the granular glands of *S. lacteus* skin. Previous studies identified the gene precursors of BF2 and F2.3S composed of a signal peptide followed by an acidic spacer peptide, which is flanked by typical Lys/Arg amino acids. The protein expression was confirmed by proteomic analysis of the secretions obtained from the SMOs culture of *S. lacteus* [25]. Here, both peptides were synthesized according to the sequences identified in previous studies [19,25]. The sequence alignment of the primary structure of the peptides buforins I and II and Parasin I with a fragment of histone H2A shows that the peptides contain almost identical sequences to the parental histone H2A, only differing in their size and region location (Figure 1). On the other hand, the amino acid alignment of frenatins derived from *S. lacteus* (frenatins 2.1, 2.2 and 2.3) shows identities close to a 100% between them. Differences among *S. lacteus* frenatin 2 members are only observed at the C-terminus, where frenatin 2.3 has an additional Gly residue. On the other hand, frenatin 2, frenatin 1.1 (*L. infrafrenata*) and frenatin 2D (*D. sardus*) have much lower identity percentages in comparison with F2.3S: 58%, 54% and 36% respectively.

The BF2 and F2.3S peptides primary structures were analyzed to identify their physicochemical properties, stability and interactions in silico. Prediction of the peptide secondary structures using the Antimicrobial Peptide Database (APD) and the PSIPRED protein sequence analysis servers indicated the tendency for α-helix structuration of both peptides (Figure 2 and Figure S1) [26,27]. Noteworthy, a previous work by NMR reported that BF2 in water adopts a random structure, while in water:trifluoroetanol (1:1) the peptide adopts a distorted helix spanning from residues Gly7 to Pro11 and a regular α-helix from Val12 to Arg20, as predicted by the PSIPRED program (Figure S1A) [7]. On the other hand, the PSIPRED server predicts for F2.3S a central α-helix spanning from Val3 to Leu15 residues (Figure S1B). The main physicochemical features of both peptides are summarized in Table 1.

The positive net charge and the amphipathic composition of the peptides confirm their potentiality for presenting antimicrobial activity. The peptide physicochemical properties are also in agreement with their capacity to interact with the anionic membranes of bacterial cells. On the other hand, some of the calculated ranks predict a differentiated behavior between the two studied peptides. A higher positive net charge is shown for BF2 in relation to F2.3S, which might explain its stronger binding affinity to nucleic acids. The hydropathicity index (GRAVY) and the W-W Hydrophobicity indicated that F2.3S might have a higher tendency to bind and cross lipid bilayers, whereas the Boman Index showed that BF2 has a higher affinity to proteins. Furthermore, the predicted stability of the peptides and calculated half-life using the APD database tool (APD, available online: http://aps.unmc.edu/AP/) suggested that the in vitro half-life and stability were higher for F2.3S than BF2, although the half-life in vivo prediction was similar for the two peptides.

### 2.2. Antibacterial and Cytotoxic Activities of BF2 and F2.3S Peptides

The peptides antibacterial activities were assessed establishing their minimum bactericidal concentrations (MBC_100_) and their 50% effective dose (ED_50_) using representative Gram-positive and Gram-negative bacteria: *Staphylococcus aureus* (ATCC502A) together with three clinical isolates, *Escherichia coli* (BL21) and two reference strains of *Pseudomonas aeruginosa* (PA01, PA14) together with two clinical isolates (see resistance profiles of clinical isolates in Table S1). The values obtained are shown in Table 2. Both peptides were effective against *S. aureus* and *E. coli* at low micromolar concentration. Nonetheless, both peptides inhibited *E. coli* growth at a lower concentration than that of *S. aureus*. Particularly, the ED_50_ for the F2.3S peptide against *E. coli* (0.23 ± 0.01) was five-fold lower than the required for *S. aureus*. On the other hand, the concentrations to inhibit the two tested reference strains of *P. aeruguinosa* were slightly higher than the ones required for *E. coli*. To note, the peptides’ efficiency against the selected clinical isolates (Table S1) was mostly reduced, in particular for BF2, which could not reach a 50% inhibitory concentration even at 100 µM (Table 2).

**Figure 3:**
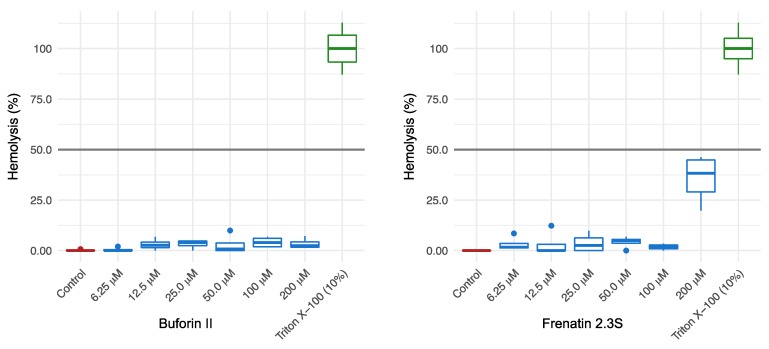
Hemolysis percentage on human erythrocytes calculated up to 200 μM for BF2 and F2.3S, as described in the methodology section. Data averaged from three replicates of two independent experiments. Values are given as mean ± SEM.

Taking into account that one of the major concerns for the use of peptides in systemic treatments is their toxicities, we evaluated the effect of both peptides against erythrocytes and primary human monocytes. The results showed that neither BF2 nor F2.3S were toxic to primary human monocytes even at a 100-fold higher concentration than their calculated antimicrobial ED_50_ (Table 2). In regard to the hemolytic activity, BF2 showed a less hemolytic effect than F2.3S. Furthermore, none of the two peptides reached a 50% hemolysis when tested up to 200 µM final concentration (Figure 3).

Minimum Bactericidal Concentrations (MBC_100_) were determined by CFU counting following plating of bacterial culture onto Petri dishes after four hours of incubation with serial peptide concentrations. The ED_50_ was calculated by ATP quantification using the BacTiter-Glo™ Microbial Cell Viability kit (Promega, Madison, WI, USA). Cell cytotoxicity was quantified using resazurin reduction in human monocytes. Fifty percent cytotoxic concentration (CC_50_) was calculated as described in Materials and methods. Data averaged from three replicates of two independent experiments. Values are given as mean ± SEM.

### 2.3. Bacterial Membrane Depolarization, Permeabilization and Agglutination Activities of BF2 and F2.3S Peptides

To characterize the antimicrobial mechanism of action by the two amphibian peptides, we evaluated their abilities to depolarize and permeabilize *E. coli* bacterial cytoplasmic membranes. Depolarization of the cytoplasmic bacterial membrane was evaluated by using the DiSC3(5) cationic membrane-permeable fluorescent dye. The dye loses fluorescence intensity in polarized membranes and becomes highly fluorescent upon membrane depolarization. The assay can detect changes in membrane ion permeability or the transmembrane potential dissipation due to a pore formation process [28]. Depolarization and leakage activity results revealed different patterns, when BF2 or F2.3S were tested. Results indicated that BF2 antibacterial action is accomplished without requiring a membrane permeabilization event [6,29]. No depolarization activity is observed even at 10-fold the effective dose for bactericidal activity. Moreover, the 50% cytoplasmic membrane leakage in *E. coli* is only achieved at a three-fold higher concentration than the ED_50_ value for its antibacterial activity (Table 2 and Table 3). In contrast, F2.3S displayed depolarization and bacterial cell leakage at lower peptide concentrations than the effective dose for *E. coli* antibacterial activity (Table 3). These finding can be related to an early membrane permeabilization and rapid leakage event process as part of the frenatin antimicrobial mechanism of action.

Moreover, in order to further characterize the mechanism of action of both BF2 and F2.3S peptides, we evaluated their potential capacity to agglutinate *E. coli* cells and calculated their minimum agglutination concentration (MAC). Hence, for the first time, we found that BF2 has the ability to agglutinate *E. coli*. Interestingly, the agglutination ability of the peptide is only effective at a higher concentration than the one required for leakage (Table 3). On the contrary, F2.3S did not have the capacity to produce agglutination in *E. coli* even at the maximum concentration tested (5 μM).

### 2.4. Leakage and Agglutination Activities on Phospholipid Liposomes by BF2 and F2.3S Peptides

After defining the peptide antibacterial activity on *E. coli* cells, we further characterized their mechanism of action on model membranes. Large unilamellar vesicles (LUVs) were prepared using a mixture of neutral/anionic phospholipids (DOPC:DOPG). Liposome leakage assays were carried out to evaluate the membrane disruption capacity of the peptides. Activity was monitored by tracing the increase of ANTS/DPX fluorescence, after mixing the loaded liposomes with the given peptide (Table 3). Results highlighted the different mechanism of action of both peptides. Whereas BF2 could not trigger the vesicle leakage at its antimicrobial effective dose, F2.3S was able to release the LUVs content at the same concentration range required for its antimicrobial activity. In addition, liposome agglutination was determined by dynamic light scattering (DLS). Changes of the liposome population were monitored upon interaction with BF2 and F2.3S. The results showed that BF2 promoted the agglutination of the lipid vesicles, whereas F2.3S was not able to agglutinate them. These results reproduced the peptides’ observed pattern on *E. coli* cultures.

### 2.5. Internalization into Bacterial Cells and In Vitro DNA Interaction by BF2 and F2.3S Peptides

**Figure 4:**
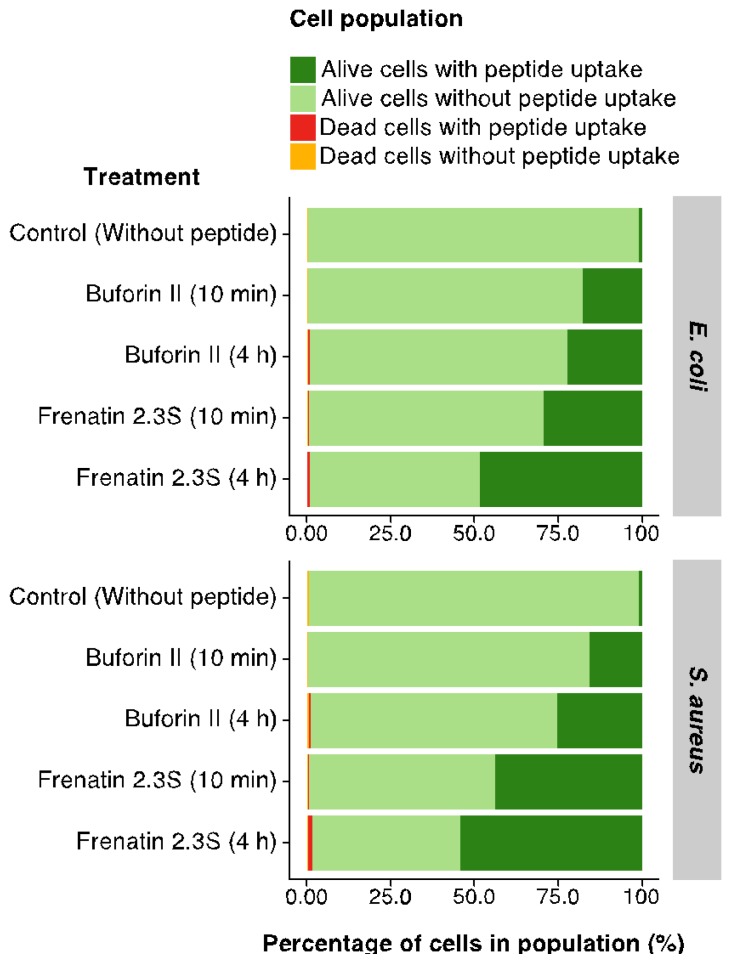
Percentage of *E. coli* and *S. aureus* cells distribution after incubation with 0.1 µM of FITC-labeled BF2 and F2.3S peptides. Cells were gated by Forward scatter (FSC)/Side scatter (SSC) using fluorescence-assisted cell sorting analysis. Additionally, the incubation mixture was treated with PI to identify the dead cell population.

Following this, in order to further explore the diverse antibacterial mechanism of actions of BF2 and F2.3S peptides, we determined the peptide bacterial cell internalization activities. We performed FACS analysis in order to analyze the internalization of the FITC labeled peptides into *E. coli* and *S. aureus* cells. The results showed the cell distribution before and after peptides incubation. A fluorescence displacement confirmed the internalization within bacterial cells of both BF2 and F2.3S labeled probes at sublethal concentrations after 10 min of incubation (Figure S2). After corroborating the internalization of the peptides, the labeled peptide distribution was evaluated by FACS. Live/dead cell population was analyzed by monitoring the influx of propidium iodide (PI) at 10 min and 4 h of incubation. The distribution of live and dead cell subpopulations together with the labeled peptide distribution is illustrated below (Figure 4 and Figure S3). Results confirmed the peptide uptake at a non-lethal concentration, where the bacterial membrane integrity was not significantly compromised.

**Figure 5:**
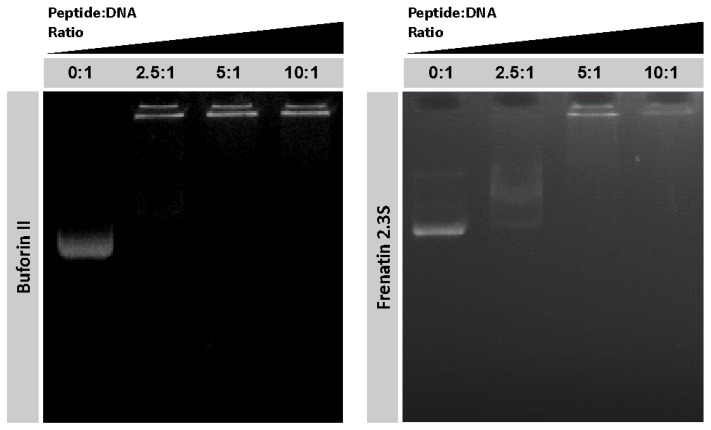
Gel retardation assay. Binding of DNA was assayed by the inhibitory effect of the peptides on migration of DNA. Different amounts of peptides were incubated with 200 ng of pET28 plasmid DNA in 20 µL of binding buffer at room temperature during 20 min and subjected to electrophoresis on a 1.0% agarose gel. The first lane corresponds to negative control without peptide.

To explore potential intracellular targets of both peptides we assayed their ability to bind DNA in vitro. DNA binding is a singular characteristic exhibited by the histone-derived antimicrobial peptides [8]. By using a gel retardation assay, we confirmed the DNA binding activity of BF2 and demonstrated that F2.3S also displays this capacity (Figure 5). Both peptides displayed a high DNA-binding activity. However, the F2.3S DNA retardation activity was slightly lower at the first tested peptide/DNA ratio.

### 2.6. Analysis of Bacterial Gene Expression Profile Pattern Induced by Peptide Incubation

**Figure 6:**
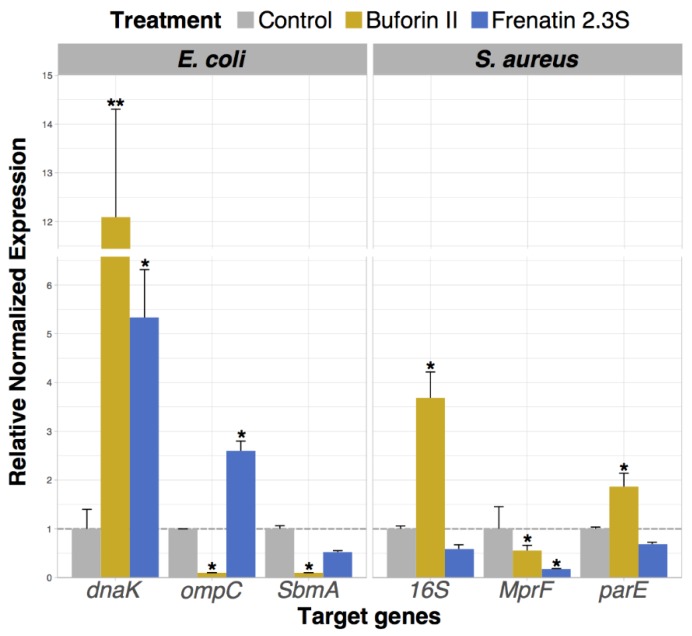
Gene expression profile in control bacterial cells and following treatment with 0.1 μM of BF2 and F2.3S during 30 min of incubation. Significant values are represented when comparing the normalized values with the GADPH expression level, which is indicated with a broken line. Significant differences respect to untreated control cells are indicated with an asterisk (*p* < 0.05).

After confirming the peptide cellular uptake, we aimed to determine the transcription patterns of certain genes in *E. coli* and *S. aureus* cells after exposition with a sublethal concentration (0.1 μM) of each peptide (Figure 6). Genes related to bacterial cellular response to stress and the development of resistance mechanisms were selected to evaluate the peptide effect on bacterial cells. Ampicillin was assayed as a positive control (results not shown). The housekeeping *GAPDH* gene served as a control. The genes evaluated for *E. coli* were *DnaK*, *ompC* and *smbA* and *16S*, *parE* and *MprF* for *S. aureus*. The results showed that the presence of BF2 increased the gene expression of the chaperone of the toposiomerase *parE* and the ribosome subunit 16S On the other hand, the expression of the porin *ompC*, the *SbmA* transporter and the *MprF* lysyl transferase were downregulated after the treatment with BF2 [30,31]. On its side, *E. coli* and *S. aureus* exposure to F2.3S induced a significantly distinct expression pattern. F2.3S, like BF2, induced the upregulation of the chaperone *DnaK* and the downregulation of the *SbmA* membrane transporter, a characteristic trait of cell stress inducers. However, F2.3S did not induce significant changes in *16S*, *MprF* and *parE* expression levels.

## 3. Discussion

The increasing emergence of bacteria resistance to conventional antibiotics is a worldwide health threat; thus, the growing interest of the pharmaceutical industry in alternative antimicrobial agents. Amphibian AMPs offer attractive antimicrobial features related with their physicochemical properties and multifaceted mechanisms of action, which facilitate a rapid and wide-spectrum bactericidal action. We report here, for the first time, the presence of BF2 in secretions of skin micro-organs of *S. lacteus*. Besides, in this work we have characterized for the first time a novel frenatin peptide (F2.3S) that was recently discovered in SMOs of *S. lacteus* in our laboratory [19].

We have characterized here both BF2 and F2.3S on Gram-negative and Gram-positive cell cultures and model membranes. The two AMPs expressed in SMOs of *S. lacteus* [19,25] are endowed with cationic and amphipathic properties (Table 1). In silico AMPs’ physicochemical analysis is considered as an important predictor to define their antibacterial potency and putative mechanism of action. In accordance with the in silico results, the higher net charge of BF2 (+6) in comparison with F2.3S (+1), leads to an enhanced electrostatic interaction between the peptide and bacterial anionic membranes [32,33,34]. This is considered the first step to initiate the translocation of peptides across the bacterial membrane [12]. Moreover, the in silico predicted results suggest that BF2 may have a high affinity to other proteins, as indicated by its Boman index (Table 1). This could indicate, that in vivo this peptide can form complex associations with other peptides to enhance its activity and stabilize its structure [32]. This result might be concomitant to the predicted stability of BF2 in vivo, in contrast to the short half-life calculated for in vitro conditions. On the other hand, F2.3S in silico predictions indicate its tendency to interact with membranes, based on the W-W interfacial hydrophobicity scale and the high hydrophobicity ratio (47%) (Table 1). This result agrees with the well-established correlation between the high hydrophobicity of many AMPs and their capacity to disturb membranes [33]. In addition, a common feature observed in membrane-active peptides is their capability to directly disturb the bilayer integrity, either by the creation of local damage, massive disruption or pore formation [32,34]. In contrast to BF2, a higher in vitro stability is predicted for F2.3S. 

Following, the two peptide activities were evaluated on Gram-negative and Gram-positive species. BF2 inhibited the growth of both Gram-negative and Gram-positive bacteria at remarkably low micromolar concentrations (Table 2). As reported previously for BF2 peptides purified from other species [9,10,11,12,13,14], our results highlight the BF2 elevated bactericidal activity. In regard to F2.3S and as predicted by in silico analysis, this peptide also presented a potent antibacterial activity against both *E. coli* and *S. aureus* reference cultures (Table 2). Moreover, along with their high and potential spectrum antibacterial capability, the new molecules must comply a low toxicity for human cells before considering their medical use [1]. Our findings discard any significant toxicity of BF2 and F2.3S on human primary monocytes at the highest tested concentration. In addition, the hemolysis rate of both peptides was lower than 50% (Figure 3) at more than 50-fold concentration its antimicrobial ED_50_, ensuring a suitable therapeutic index. Notwithstanding, F2.3 shows a comparatively higher hemolysis rate in comparison to BF2. This difference might probably be due to the high hydrophobicity of F2.3S peptide versus BF2, as observed for other peptides [35].

AMPs have different strategies to carry out a rapid and effective removal of microbial infection. Among the major biophysical indicators of AMPs mechanism of action we find membrane depolarization and leakage, as well as cellular agglutination, cellular internalization and nucleic acid binding [4,36,37]. In this study, first we evaluated the membrane depolarization and permeabilization capacity of both peptides in *E. coli* cultures. As previously reported, BF2 is able to enter into the cytoplasmic membrane without generating significant depolarization and/or cellular leakage (Table 3). This pattern is consistent with the currently accepted mechanism of BF2 action that establishes its ability to translocate into bacteria without disrupting the membrane [9,10,11,12,13,14]. Our findings unveil for the first time the capacity of BF2 to agglutinate *E. coli* cells and liposomes when increasing the peptide concentration above its effective antimicrobial concentration. The result for BF2 is in agreement with other reports that highlight the importance of electrostatic interaction as a part of the peptides aggregation mechanism of action [34,38]. Interestingly, it is frequently observed that the aggregation activity does not participate in the initial stage of the antimicrobial mechanism, as reported for example for the Dermaseptin S9 peptide or the human secretory RNase derived peptides [39,40]. This agrees with our findings that show that the BF2 concentrations at which the cellular aggregation takes place are well above its ED_50_ value. Nonetheless, we conceive that the BF2 cellular agglutination activity is indeed part of its mechanism of action. Some agglutinating peptides can contribute to prevent the spread of infection by facilitating the phagocytosis of bacteria clumps [41]. In addition, BF2 promotion of cell agglutination might be enhanced by its union by electrostatic interactions to the lipopolysaccharides (LPSs) at the outer membrane of Gram-negative bacteria and reduce the endotoxin circulating free levels, as reported for other AMPs [42,43,44,45].

On the other hand, we report here for the first time the antibacterial mechanism of F2.3S. The peptide has a membrane destabilization mechanism that involves the rapid depolarization of the membrane and its further destabilization. On the contrary, the agglutination activity of BF2 is not shared by F2.3S. In any case, both peptides can translocate into the cytosol and interact with nucleic acids. Cell internalization and DNA binding assays were performed taking into account the previously described BF2 capacity to penetrate membranes without lysing bacteria. Once inside, BF2 bounds tightly to nucleic acids, inhibiting macromolecular processes of the cell [46]. Interestingly, our results showed that F2.3S is also able to inhibit the migration of DNA in a gel electrophoresis assay, although this activity was only evidenced when doubling the peptide/DNA ratio respect to BF2 (Figure 5). The lower positive net charge observed for F2.3S in relation to BF2 could explain the observed difference. To note, a prediction model of buforin interaction with DNA indicates a merely electrostatic interaction associated to the Arg content and discards any base specific binding mode [47]. In addition, we confirmed the cellular uptake of both peptides at sub-lethal concentrations. The flow cytometry results corroborate that BF2 is translocated into the bacterial cytoplasm. The process was also observed for F2.3S, showing even a higher percentage of the cell population that incorporated the labeled peptide, in comparison to the treated cells with BF2 (Figure 4). Therefore, F2.3S at sublethal concentrations could be internalized and can target intracellular molecules such as DNA.

Following, we evaluated the putative intracellular effect of BF2 and F2.3S in *E. coli* and *S. aureus* by monitoring the expression pattern of selected genes related to bacterial stress conditions. The results showed that in *E. coli*, the *DnaK* gene was upregulated after the exposition of the cells to BF2 and F2.3S, as observed for the ampicillin positive control. This finding demonstrates that BF2 and F2.3S at a membrane non-lytic concentration alter the bacterial cell gene expression profile. Interestingly, *DnaK* is one of the most abundant constitutively expressed and stress-inducible chaperones in the *E. coli* cytosol but it is not essential under non-stress conditions [48]. Conversely, the porin *ompC* gene was downregulated by BF2 and upregulated by F2.3S. According to the bibliography, the *E. coli ompC* gene expression is usually altered in response to different antibiotics exposure [49,50]. We find in the literature, together with other cell penetrating peptides, reports of BF2 exerting its antimicrobial action by interfering with key cellular roles, such as cell wall synthesis and protein-folding assisted by chaperones [51]. Similarly to BF2, other studies have reported that *E. coli* exposed to the AMPs, such as Esculetin, diminished the expression of the *ompC* gene [51]. In regard to the *sbmA* gene of *E. coli*, we observe a mild reduction of its expression in the presence of both peptides, as observed for the ampicillin positive control. This gene encodes a transmembrane protein transporter [52]. The downregulation of the *SbmA* can block the transport of antibiotics into the cell cytoplasm and can represent a bacterial resistance mechanism. To note, the bacterial downregulation of *ompC* and *SbmA* genes that are related to peptide uptake might represent a mechanism of protection against AMPs [49,53]. For example, mutant strains lacking the *OmpC* porin acquired antibiotic resistance [54]. Significant changes in the gene expression pattern in *S. aureus*, after exposition with BF2 and F2.3S, were also registered. Specifically, the expression of the *16S* and *parE* genes was upregulated in the presence of BF2, whereas the expression of the *MprF* gene was only slightly altered for the treated cells with both peptides. We hypothesize that transcription upregulation of 16S and the topoisomerase *parE* upon bacterial incubation with BF2 can constitute an adaptation response mechanism to the peptide binding to nucleic acids. On the other hand, modulation of the *MprF* gene, involved in the synthesis of the bacterial wall exposed positively charged LPG has been related to bacterial evasion of cationic peptides action [55,56]. However, the present results suggest that the assayed conditions were not sufficient to induce a significant bacterial response to BF2 and F2.3S exposure. Overall, our data reveal for the first time the ability of BF2 and F2.3 peptides to induce the bacterial stress response and alter specific genes involved in bacterial resistance mechanisms [57]. We can conclude that BF2 exposure mostly shows a prominent shift of the bacteria gene transcription profile pattern associated to cellular stress, e.g., increase expression of *DnaK* chaperone, *parE* DNA topoisomerase IV and 16S rRNA. On the other hand, the gene expression pattern is less altered by F2.3S exposure. However, the increase of *DnaK* following F2.3S exposure indicates to some extent the activation of bacterial cellular stress processes. The reduced response related to bacterial resistance markers observed for F2.3S might be explained by its different mechanism of action. The higher antimicrobial activity and more prominent membrane destabilization activity of F2.3S might impede the emergence of bacterial response processes. 

Overall our results suggest that BF2 and F2.3S identified in SMOs of *S. lacteus* can exert diverse bacteria killing strategies at an effective concentration range that is potentially non-toxic to human host cells. We have confirmed that BF2 mechanism of action involves translocation and intracellular targets. In this regard, we evidenced that BF2 altered the expression of key survival genes from the bacteria. In addition, we found that bacterial agglutination can also participate in the BF2 action against Gram-negative bacteria. We hypothesize that this mechanism can facilitate the bacteria removal from the infection focus and might reduce the LPS blood circulating levels and thereby have beneficial anti-endotoxin properties. On the other hand, F2.3S can also achieve its translocation into bacterial cells at sublethal concentrations, and shows an even higher antimicrobial activity in comparison to BF2. The results suggest that F2.3S can combine its membrane destabilizing action together with an intracellular targeting of key cellular functions. Thereby, both frog AMPs can undertake distinct mechanisms of action dependent on their relative effective concentration [34,58,59,60,61]. Therefore, the two peptides purified from *S. lacteus* constitute another example of secreted AMP with multifunctional mode of action [56,59,62]. The present work corroborates that frog skin secretory micro-organs represent a continuing source of novel peptides with multifaceted mechanisms of action. Taking into consideration that SMOs mimic an open injured tissue [63], we are confident that frog secreted AMPs would be regarded as attractive lead molecules to develop novel antibiotic therapies. Indeed, the first AMP selected for Phase III clinical trials to treat wound injuries, *Pexiganan*, is a short synthetic analogue of magainin, an AMP secreted by the frog *Xenopus laevis* [64]. Moreover, recent in vivo studies in mice models confirmed the wound healing properties of frog AMPs [65]. We propose here BF2 and F2.3S as potential lead candidates to assist in the design of novel antimicrobial agents against superbugs that have developed multiple strategies of resistance.

## 4. Materials and Methods

### 4.1. In Silico Analysis

In silico analysis of the BF2 and F2.3S general peptide characteristics, stability related features, sequence similarities with other related peptides, as well as the stability in vitro and in vivo were analyzed with a broad repertoire of databases and bioinformatics tools. Using HELIQUEST the alpha helix conformation and the physicochemical properties of these peptides was explored. The “APD3: Antimicrobial Peptide Calculator and Predictor” tool of the Antimicrobial Peptide Database (APD) [26] was used to identify the following general characteristics of antimicrobial peptides: net charge, hydrophobic and hydropathy (GRAVY) ratio, potential capacity to attach to other proteins (Boman Index), alpha helices structuration or sequence specific amino-acids enrichment (Table 1). The *Membrane Protein Explorer* (MPEx) allowed us to determine the potential of the peptides to bind to membrane bilayers [66]. The *ProtParam Tool* was used to identify the stability features of the two peptides, allowing recognition of their suitability to remain stable in specific environments [67]. Based on the N-terminal amino acid of each peptide, the half-life in vitro and in vivo was predicted along with the aliphatic index and classification of the stability of the peptide. Likewise, the “HLP: Web server for predicting half-life of peptides in intestine-like environment” permitted the prediction of the half-life and stability of the new peptides in an intestine-like, proteolytic environment [68].

### 4.2. Peptide Synthesis

Previous work from our laboratory identified precursors from BF2 and F2.3S secreted by *S. lacteus* skin micro-organs [19,25]. Based on those sequences we synthesized and purified BF2 (TRSSRAGLQFPVGRVHRLLRK) and F2.3S (GLVGTLLGHIGKAILGG) (Accession no.AGB51284.1). The peptide synthesis was performed by GL Biochem Shanghai (Shanghai, China) and the Peptide Synthesis Facility at Proteomic Unit, Prof. David Andreu laboratory, Universitat Pompeu Fabra (UPF), Spain. Purification was performed by HPLC (>95%) on a Vydac C_18_ (2.2 cm × 25 cm) and Luna C_18_ (4.6 × 50 cm) columns. The unlabeled and FITC-labeled peptides were purified by Luna C18 with a linear gradient of 0.036% TFA in MeCN into 0.045% TFA in H_2_O at a flow rate 1 mL/min. The peptide masses were confirmed by liquid chromatograph-mass spectrometry (LC-MS) using a 2010EV Shimadzu instrument (Kyoto, Japan). Purification profiles and MS spectra are shown in Figure S4.

### 4.3. Minimum Bactericidal Concentration

Antimicrobial activity was calculated as the 100% minimum bactericidal concentration (MBC_100_), defined as the lowest peptide concentration that completely eradicated microbial growth. The MBC of each peptide was determined as described previously [69,70]. Bacteria were incubated at 37 °C overnight in Luria-Bertani broth (LB) and diluted to approximately 5 × 10^5^ CFU/mL. The strains used in this study corresponded to *Escherichia coli* BL21 from Novagen (Madison, WI, USA), *Staphylococcus aureus* (ATCC502A) obtained from the *Colección Española de Cultivos tipo* (CECT), Universidad de Valencia, Spain, *Pseudomonas aeruginosa* (ATCC^®^ 15692, PAO1), from the American Type Culture Collection (ATCC; Rockville, MD, USA) and *P. aeruginosa* PA14 were used as reference strains. Three clinical isolates of *S. aureus*, MRSA (34026, 36055 and 39413) and two clinical isolates of *P. aeruginosa* (M8C1 and M18C1) were obtained by the kind donation of the *Centro de Investigaciones Microbiológicas*, CIMIC (Universidad de los Andes, Bogotá, Colombia). In each assay, peptides were serially diluted from 20 to 0.1 µM final concentration in Hepes 20 mM, 0.1 M NaCl, pH 7.5 buffer and added to 100 µL aliquots of the bacterial dilution and incubated for 4 h at 37 °C. Subsequently, samples were plated onto Petri dishes and incubated at 37 °C overnight. MBC values were determined as a function of the total removal of CFU by the peptide from two independent experiments performed in triplicate.

### 4.4. Cell Viability Assay by ATP Quantification

Antibacterial activity was assayed by following the cell viability of *E. coli* and *S. aureus*, using the BacTiter-Glo™ Microbial Cell Viability kit (Promega), which measures the number of viable bacterial cells, by ATP quantification. ATP, as an indicator of metabolically active cells, is indirectly measured by a coupled luminescence detection assay. The luminescent signal is proportional to the amount of ATP required for the conversion of luciferin into oxyluciferin in the presence of luciferase. Briefly, an overnight culture of *E. coli* was inoculated in a fresh LB to reach logarithmic phase OD_600_ of 0.2, washed and resuspended in Hepes 20 mM pH 7.5, 0.1 M NaCl. Peptide concentration was assayed from 100 to 0.1 µM. *E. coli* viability was followed after 4 h of incubation at 37 °C. 50 µL of incubation culture were mixed with 50 µL of BacTiter-Glo™ reagent in a microtiter plate following the manufacturer instructions and incubated at room temperature for 10 min. Luminescence was read on a Victor3 plate reader (PerkinElmer, Waltham, MA, USA) with a 1-s integration time. Fifty percent effective dose concentrations (ED_50_) were calculated by fitting the data to a dose–response curve with Prism6^®^.

### 4.5. Hemolysis Assay

The hemolytic activity of the peptides was determined by a previous method [71]. Briefly, 2 × 10^7^ erythrocytes from healthy human blood were washed three times with 150 mM of NaCl and then replaced with 1 mL of PBS at pH 7.4. Serially diluted concentrations of BF2 and F2.3S were prepared up to a maximum final concentration of 200 μM. The hemolytic assay was performed in flat-bottomed 96-well microtiter trays, 10 μL of each peptide concentration and 190 μL of the diluted red blood cells were incubated for 1 h at 37 °C. After incubation, the assay was centrifuged and the absorbance (450 nm) of each resuspended pellet was measured on a microplate absorbance spectrophotometer (Bio-Rad, Philadelphia, PA, USA). The positive control was an erythrocyte suspension incubated in 10% of Triton (100% hemolysis), and the negative control was an erythrocyte suspension incubated in PBS 1× (0% hemolysis).

### 4.6. Cytotoxicity Assay in Human Monocytes

Human monocytes were isolated from peripheral human blood by Ficoll–Hypaque density gradient (Sigma Chemical Co., St. Louis, MO, USA). The buffy coats were washed with 5 mL of saline solution, pH 7.5 and placed in Petri dishes with RPMI 1640 medium (Gibco BRL-life Technologies Inc., Grand Island, NY, USA), 5% fetal bovine serum (Microgen, Bogotá, Colombia), and after 24 h incubation at 37 °C the adherent cells were recovered using a scraper. The cell viability was determined using trypan blue. Human monocytes (4 × 10^5^ cells/well) and BF2 and F2.3S peptides serially diluted up to 100 µM were cultured in 96 well plates for 72 h at 37 °C. Finally, the resazurin reduction assay was used to evaluate the cytotoxicity of these peptides at 595 nm. Addition of resazurin (Sigma-Aldrich, St. Louis, MO, USA) was added at 44 µM final concentration. The cytotoxic concentration 50, CC_50_, was taken as the mean concentration of peptide producing 50% cell death in two independent experiments and was calculated by non-linear regression analysis using GraphPad Prism software version 6.0 (GraphPad Software, San Diego, CA, USA).

### 4.7. Bacterial Cell Membrane Depolarization Assay

Membrane depolarization was assayed by monitoring the DiSC_3_(5) fluorescence intensity in response to changes in transmembrane potential as described previously [72]. *E. coli* cells were grown at 37 °C to the mid-exponential phase and resuspended in 5 mM Hepes-KOH, 20 mM glucose and 100 mM KCl at pH 7.2 until OD_600_ of 0.05 was reached. DiSC_3_(5) was added to a final concentration of 0.4 μM. Changes in the fluorescence for alteration of the cytoplasmic membrane potential were continuously monitored at 20 °C at an excitation wavelength of 620 nm and an emission wavelength of 670 nm. When the dye uptake was maximal, as indicated by a stable reduction in the fluorescence as a result of quenching of the accumulated dye within the membrane, the protein diluted in 5 mM Hepes-KOH buffer at pH 7.2 was added at a final peptide concentration from 0.1 to 5 µM. All conditions were assayed in duplicate. The time required to reach a stabilized maximum fluorescence reading was recorded for each condition, and the time required to achieve half of total membrane depolarization was estimated as effective dose values (ED_50_).

### 4.8. Bacterial Cell Leakage Assay

Bacterial cell leakage was evaluated using the SYTOX Green assay. *E. coli* cultures were grown to mid-exponential growth phase (OD_600_ ~ 0.2) in LB medium and then centrifuged (5000× *g* for 2 min at 25 °C), washed and resuspended in phosphate buffer. Cell suspensions for OD_600_ ~ 0.05 were incubated with 5 µM SYTOX Green (Invitrogen, Carlsbad, CA, USA) during 15 min in the dark before the influx assay. At 2–4 min after initiating data collection peptide concentrations from 0.625 to 5 μM were added to the cell suspension, and the increase in SYTOX Green fluorescence was measured (485 and 520 nm excitation and emission wavelengths respectively) for 40 min in a Cary Eclipse spectrofluorimeter, Agilent Technologies, Madrid, Spain. Maximum fluorescence was that resulting from cell lysis with Triton^TM^ X-100 (Sigma-Aldrich).

### 4.9. Minimum Agglutination Concentration (MAC)

For determination of minimum agglutination concentration (MAC) values, bacterial cells were grown at 37 °C to mid-exponential phase (OD_600_ = 0.6), centrifuged at 5000× *g* for 2 min, and resuspended in Tris-HCl buffer, 0.1 M NaCl, pH 7.5 to give an absorbance of 0.2 at 600 nm. A 100 µL aliquot of the bacterial suspension was incubated with the peptide at various (0.01–10 µM) concentrations at 37 °C for 1 h. Agglutination behavior was observed by visual inspection using a 50× stereoscope microscope and the MAC value was expressed as described [45].

### 4.10. Large Unilamellar Vesicle (LUV) Liposome Preparation

LUVs containing DOPC/DOPG (3:2 molar ratio) of a defined size (approximately 100 nm) were prepared as described previously [73]. LUVs were obtained from a vacuum-drying lipid chloroform solution by extrusion through 100 nm polycarbonate membranes. The lipid suspension was frozen and thawed ten times before extrusion. A 1 mM stock solution of liposome suspension in 10 mM phosphate buffer and 100 mM NaCl (pH 7.4) was prepared.

### 4.11. Liposome Membrane Leakage Activity

Membrane leakage activity was assessed by ANTS/DPX (8-aminonaphthalene-1,3,6-trisulfonic acid disodium salt/p-xylenebispyridinium bromide) as previously [74]. Large unilamellar vesicles of dioleoylphosphatidylcholine: dioleoylphosphatidylglycerol (3:2 molar ratio), containing 12.5 mM ANTS and 45 mM DPX in 20 mM NaCl, 10 mM Tris/HCl, pH 7.5 were prepared. The ANTS/DPX liposome stock suspension was diluted to 30 μM and incubated at 37 °C with protein/peptide, serially diluted from 20 μM to 0.1 μM in a microtiter plate. Fluorescence measurements were performed on a Victor3 plate reader (PerkinElmer, Waltham, MA, USA). ED_50_ values were calculated by fitting the data to a dose–response curve with Prism6^®^.

### 4.12. Dynamic Light Scattering (DLS)

Liposome agglutination was analyzed by DLS (dynamic light scattering) using a Malvern 4700 photon correlation spectrometer (Malvern Instruments, Malvern, UK). An argon laser (λ = 488 nm) was used to cover the wide size range involved. Hydrodynamic radius measurements were always carried out at a reading scattering angle of 90°. From the intensity measurements recorded, data were processed by the CONTIN software (Malvern), and the hydrodynamic diameter, the polydispersity index and the total number of counts were calculated. The incubation buffer was 10 mM Tris/HCl and 100 mM NaCl (pH 7.4). Measurements were performed at 25 °C, at 30 µM final liposome concentration and 0.1–2 µM BF2 and F2.3S peptide concentrations.

### 4.13. Fluorescence-Activated Cell Sorter (FACS) Assay

Bacterial cell overnight culture was inoculated in a fresh LB to reach logarithmic phase OD_600_ of 0.2. A 500 µL aliquot of the bacterial suspension was incubated with 0.5 µM of each peptide for 10 min. After incubation and subsequently washes with 1× PBS at least thrice to remove presence of FITC-labeled peptide in the medium, 20,000 cells were subjected to FACS analysis using a FACSCalibur cytometer (BD Biosciences, Franklin Lakes, NJ, USA). The peptide bacterial uptake was assayed using 0.10 µM and 0.5 µM of each peptide at the first 10 min and after 4 h of peptide incubation. The histogram represents the peptide fluorescence into the bacterial cells (*X* axis) and cellular count (*Y* axis). The influx of propidium iodide (PI), a DNA-staining fluorescent probe, and FITC-labeled peptides BF2 and F2.3S into bacterial cells (*E. coli* and *S. aureus*) was investigated by using a dual laser fluorescence- activated cell sorter and represented by percentage of cell distribution using R software R (Foundation for Statistical Computing, Vienna, AT, USA) [75].

### 4.14. DNA Binding Assay

The binding of peptides to DNA was examined by a gel retardation assay as described previously [7,10]. Briefly, 200 ng of pET28 plasmid DNA was incubated with increasing concentrations of BF2 and F2.3S in 20 μL of binding buffer (5% glycerol, 10 mM Tris-HCl (pH 8.0), 1 mM EDTA, 1 mM DTT, 20 mM KCl and 50 ng/mL BSA) at room temperature for 20 min and subjected to electrophoresis on a 1.0% agarose gel. DNA bands were visualized by ethidium bromide staining. The peptide-to-DNA weight ratios were 0:1, 2.5:1, 5:1 and 10:1 respectively. The running conditions were established according to [76].

### 4.15. Gene Expression Analysis

The bacterial gene expression of proteins implicated in stress pathways and bacterial resistance response was evaluated following incubation with sub-lethal concentrations of the peptides. The following genes were selected: *DnaK* gene encoding for a chaperone, *ompC* gene encoding for a protein porin; *sbmA* encoding for an integral inner membrane protein that participates in an ABC transporter, *parE* encoding for a DNA topoisomerase IV and *MprF* for a lysyl transferase that ensures the synthesis of lysylphosphatidylglycerol (LPG). Total bacterial RNA was extracted using the mirVAna™ Isolation Kit, which integrates an organic extraction and solid-phase extraction to obtain high yields of ultra-pure, high quality RNA. Bacterial cells culture (1.5 mL) at log-phase (OD_600_ of 0.2) were incubated using 0.1 μM of BF2 and F2.3S peptides during 30 min. Ampicillin was used at the same concentration as a positive control. After incubation cells were sedimented and resuspended in lysis buffer. RNA isolation was done according to manufacturer’s instructions. The amount of RNA extracted was quantified using Quantus™ Fluorometer (Promega). Approximately 150 ng/μL was obtained per culture sample. Complementary DNA (cDNA) synthesis is the first step of the two-step quantitative reverse transcriptase Polymerase Chain Reaction (RT-qPCR). cDNA was generated by the enzyme reverse transcriptase (RT). An aliquot of 1000 ng of total RNA was used to synthesize cDNA according to the manufacture instruction of iScript™ cDNA Synthesis Kit.

### 4.16. Real Time Quantitative PCR Polymerase Chain Reaction (RT-qPCR)

Gene expression of *Escherichia coli* and *Staphylococcus aureus* after peptide incubation was determined by Real Time Quantitative Polymerase Chain Reaction (RT-qPCR). The amount of amplified product was measured at each PCR cycle by using fluorescent probes and reading the signal emitted from the dyes while performing the thermal cycling. 1 μL of cDNA (5 ng) was mixed with 2 μL of RNase free water, 5 μL of iTaq Universal Master SYBR green Supermix, 1 μL of each primer of interest (500 nM), for a total volume of 10 μL per reaction. The genes studied in *E. coli* cells correspond to *DnaK*, *ompC*, *SbmA* and 16S *MprF*, *parE* for *S. aureus*. GAPDH was used as housekeeping for date normalization in the qPCR assays. Gene expression level was analyzed using a comparative quantification method ΔCq. This method assesses the fold change compared to a calibrator sample (control or group of reference). Counts for genes of interest were adjusted in relation to a reference (housekeeper) gene GAPDH. Reactions were subjected to 60 °C for 1 min and 95 °C for 10 min, followed by 40 cycles of 95 °C for 15 s and 60 °C for 1 min. Each sample was analyzed in triplicate. Data analysis was performed using Bio-Rad CFX Manager. The figure of gene level expression was designed by R project [75].

### 4.17. Statistical Analysis

Statistical analyses were performed using Prism6^®^ by one-way ANOVA. The results are from three independent experiments performed on different days. A *p* value < 0.05 was considered statistically significant.

## 5. Conclusions

Our laboratory identified for the first time the presence of buforin BF2 and frenatin F2.3S in the skin micro-organs (SMOs) from the Orinoco lime treefrog (*S. lacteus*). Here, we have characterized both antimicrobial peptide properties. Evaluation of BF2 on bacteria cultures and model membranes confirmed its previously reported non-membranolytic mechanism of action. Following intracellular translocation, BF2 can interact with DNA and alter the bacteria transcription profile. Complementarily, we found for the first time that BF2 can also induce the agglutination of bacterial cells. On the other hand, this is the first report of F2.3S antimicrobial properties. Frenatins constitute a small peptide family very poorly studied till now that is attracting particular interest because of its multifaceted properties. We demonstrate here that F2.3S displays a high antimicrobial activity for both Gram-negative and Gram-positive species. The peptide can also translocate into the bacterial cytosol, bind to nucleic acids and induce the expression of genes related to cellular stress. Besides, F2.3S can induce membrane depolarization and leakage but cannot trigger the bacterial cell agglutination. In summary, our study demonstrates that the mechanism of F2.3S and BF2 identified in the SMOs of *S. lacteus* involves actions both at the bacterial surface and intracellular levels, highlighting their multifunctional antimicrobial activities. In particular, the present characterization of F2.3S unveils its appealing properties for the design of lead drug candidates to develop alternative antibiotic agents.

## Figures and Tables

**Table 1:** In silico physicochemical properties of the BF2 and F2.3S peptides.

Physicochemical Properties	Peptides	
	BF2	F2.3S
Theoretical mass (Da)	2434	1570
Experimental mass (Da) ^1^	2434.88	1575.93
Net charge	+6	+1
Isoelectric point	12.44	9.07
GRAVY	−0.63	1.17
Hydrophobic ratio	33%	47%
W-W Hydrophobicity ^2^ (Kcal/mol)	7.73	5.55
Boman index (Kcal/mol)	3.34	−1.66
Stability	Unstable	Stable
Half-life in vitro (*Mammalian reticulocytes*) ^3^	7.2 h	30 h
Half-life in vivo (*Yeast*) ^3^	>20 h	>10 h
Half-life in vivo (*E. coli*) ^3^	>20 h	>10 h

^1^ Average experimental mass of synthetic peptides (see Figure S4 for HPLC chromatograms and MS spectra). ^2^ Octanol-Interface scale. ^3^ Calculated using the APD server.

**Table 2:** Antimicrobial and cytotoxic activities of BF2 and F2.3S peptides.

Biological Activity		Peptide	
		BF2	F2.3S
Gram negative	MBC_100_ (µM)	0.93	0.62
*E. coli* (*BL21*)	ED_50_ (µM)	0.33 ± 0.005	0.23 ± 0.01
	ED_50_ (µM)	1.3 ± 0.082	3.4 ± 0.062
*P. aeruginosa* (*PA01*)	ED_50_ (µM)	1.8 ± 0.005	3.1 ± 0.003
*P. aeruginosa* (*PA14*)	ED_50_ (µM)	>100	>100
*P. aeruginosa* (*M8C1*) *	ED_50_ (µM)	>100	>100
*P. aeruginosa* (*M18C1*) *			
Gram positive	MBC_100_ (µM)	1.87	1.87
*S. aureus* (*ATCC 502A*)	ED_50_ (µM)	0.51 ± 0.02	1.1 ± 0.07
	ED_50_ (µM)	6.46 ± 0.002	11.99 ± 0.001
*S.aureus* (*39413*) *	ED_50_ (µM)	>100	55.82 ± 0.005
*S.aureus* (*34026*) *	ED_50_ (µM)	>100	>100
*S.aureus* (*36055*) *			
Human monocytes	CC_50_ (µM)	>100	>100

* Clinical isolates: the antibiotic resistance profile is detailed in Table S1.

**Table 3:** Depolarization, leakage and agglutination activities toward *E. coli* cells and/or DOPC/DOPG liposomes.

Biological Activity		Peptide	
		BF2	F2.3S
Depolarization (ED_50_) (µM)	Bacteria	>5	0.1 ± 0.05
Leakage (ED_50_) (µM)	Bacteria	0.9 ± 0.1	0.1 ± 0.07
	Liposomes	>2	0.1
Agglutination (MAC) (µM)	Bacteria	1.5	>5
	Liposomes	0.5 ± 0.002	>2

The peptide concentration required to achieve half of total membrane depolarization and leakage were estimated as effective dose values (ED_50_) using the membrane-potential-sensitive DiSC_3_ dye and SYTOX Green assays respectively. Minimum agglutination values (MAC) were calculated as described in the methodology. Values are given as mean ± SEM calculated from three replicates of two independent experiments.

## Supplementary Materials

Supplementary materials can be found at http://www.mdpi.com/1422-0067/19/8/2170/s1.

## Abbreviations

APD	Antimicrobial peptide databases
AMPs	Antimicrobial peptides
ANTS	8-aminonaphthalene-1,3,6-trisulfonic acid
BF2	Buforin II
CFU	Colony forming units
DLS	Dynamic light scattering
DOPC	1,2-dioleoyl-sn-glycero-3-phosphocholine
DOPG	1,2-Dioleoyl-sn-glycero-3-phosphoglycerol
DTT	Dithiothreitol
ED50	50% effective dose
FACS	Fluorescence-activated cell sorting
F2.3S	Frenatin 2.3S
IL	Interleukin
LB	Luria-Bertani media
LPS	Lipopolysaccharide
LUV	Large unilamellar vesicle
MAC	Minimum agglutination concentration
MBC	Minimum bactericidal concentrations
MPEx	Membrane protein explorer
SMOs	Skin micro-organs
FITC	Fluorescein
TNFα	Tumor necrosis factor-alpha
