# Supplementary material for: Unveiling the Multifaceted Mechanisms of Antibacterial Activity of Buforin II and Frenatin 2.3S Peptides from Skin Micro-Organs of the Orinoco Lime Treefrog (Sphaenorhynchus lacteus)
Source: Int J Mol Sci. 2018 Jul 25;19(8):2170. doi: 10.3390/ijms19082170 (PMC6121439; doi:10.3390/ijms19082170)
Supplement: Supplementary file 1 [file ijms-19-02170-s001.zip › ijms-333445-SI.pdf]

International Journal of *Molecular Sciences*

Supplementary Materials

Unveiling the multifaceted mechanisms of antibacterial activity of Buforin II and Frenatin 2.3S peptides from skin micro-organs of the Orinoco lime treefrog (*Sphaenorhynchus lacteus*)

Carolina Muñoz-Camargo[1,2,3], Vivian A. Salazar[2,3], Laura Barrero-Guevara[2], Sandra Camargo[2,5], Angela Mosquera[3,4], Helena Groot[2,*] and Ester Boix[3,*]

[1] Department of Biomedical Engineering, Universidad de los Andes; c.munoz2016@uniandes.edu.co

[2] Laboratorio de Genética Humana, Department of Biological Sciences, Universidad de los Andes; visalazar@uniandes.edu.co; sandracamargouis@gmail.com; la.barrero1854@uniandes.edu.co; hgroot@uniandes.edu.co;

[3] Department of Biochemistry and Molecular Biology, Faculty of Biosciences, Universitat Autònoma de Barcelona; ester.boix@uab.cat

[4] Biotechnology Group, Biology Institute, Universidad de Antioquia; angela.mosquera@udea.edu.co

[5] Institute of Life Sciences, Hebrew University of Jerusalem

* Correspondence: Ester.Boix@uab.es, Tel.: 34-935812565; hgroot@uniandes.edu.co, Tel: 571-3394949 ext.2771

Supplementary Materials

Table S1. Antibiotic resistance profile of evaluated bacteria clinical isolates.

**Bacteria**	**Sensible to**	**Resistant to**
S. aureus (39413)	Ampicillin<br>Benzylpenicillin<br>Cefazolin<br>Clindamycin<br>Erythromycin<br>Oxacillin<br>Quinupristin-dalfopristin<br>Rifampin<br>Tetracycline	
S. aureus (34026)	Ceftriaxone<br>Clindamycin<br>Erythromycin<br>Gatifloxacin<br>Gentamicin<br>Levofloxacin<br>Oxacillin<br>Penicillin	Linezolid<br>Moxifloxacin<br>Quinupristin-dalfopristin<br>Tetracycline<br>Vancomycin<br>Ampicillin-sulbactam
S. aureus (36055)	Ceftizoxime<br>Clindamycin<br>Erythromycin<br>Gatifloxacin<br>Levofloxacin<br>Oxacillin	Moxifloxacin<br>Quinupristin-dalfopristin<br>Tetracycline<br>Ampicillin-sulbactam<br>Gentamicin
	Penicillin	
P. aeruginosa (M8C1)	Ampicillin	Ciprofloxacin<br>Cefotaxime<br>Gentamicin
P. aeruginosa (M18C1)	Nefloxacin<br>Ampicillin<br>Imipenen<br>Aztreonam	Cephalothin<br>Sulfatrimetropin

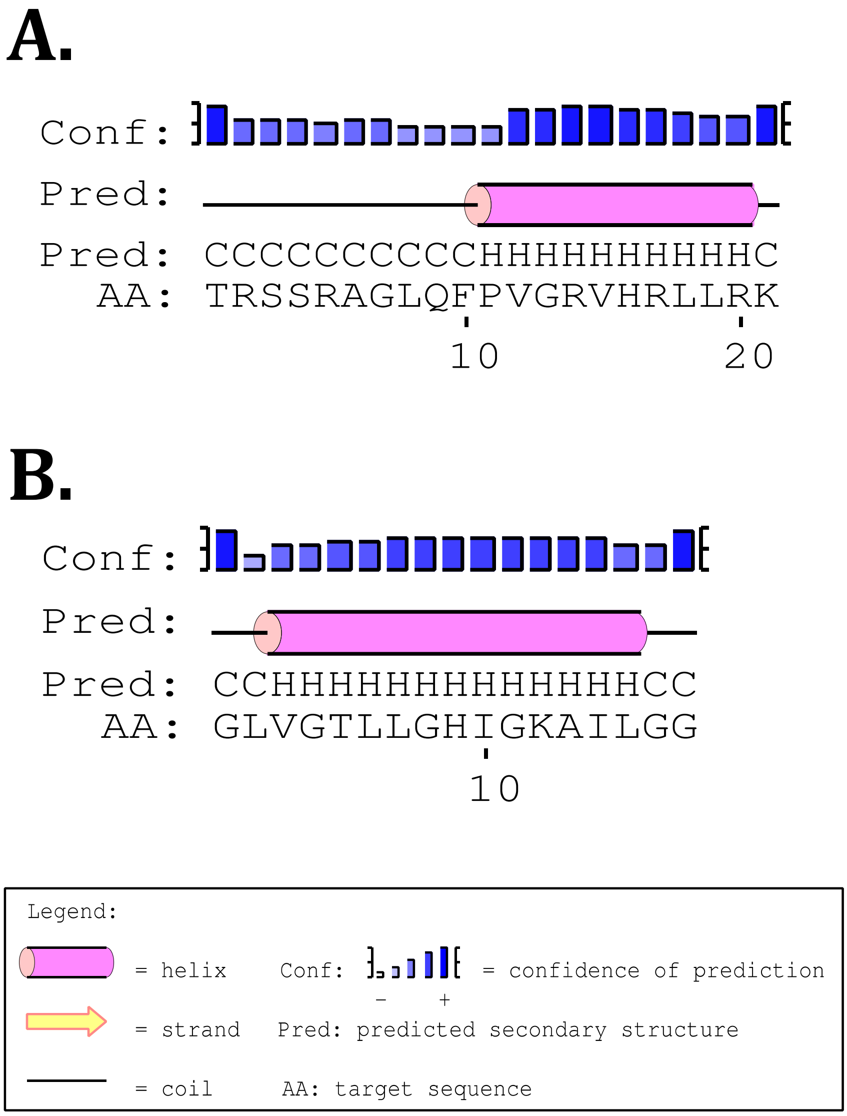

Figure S1. Secondary structure prediction of BF2 (A) and F2.3S (B) using **the** *PSIPRED* server.

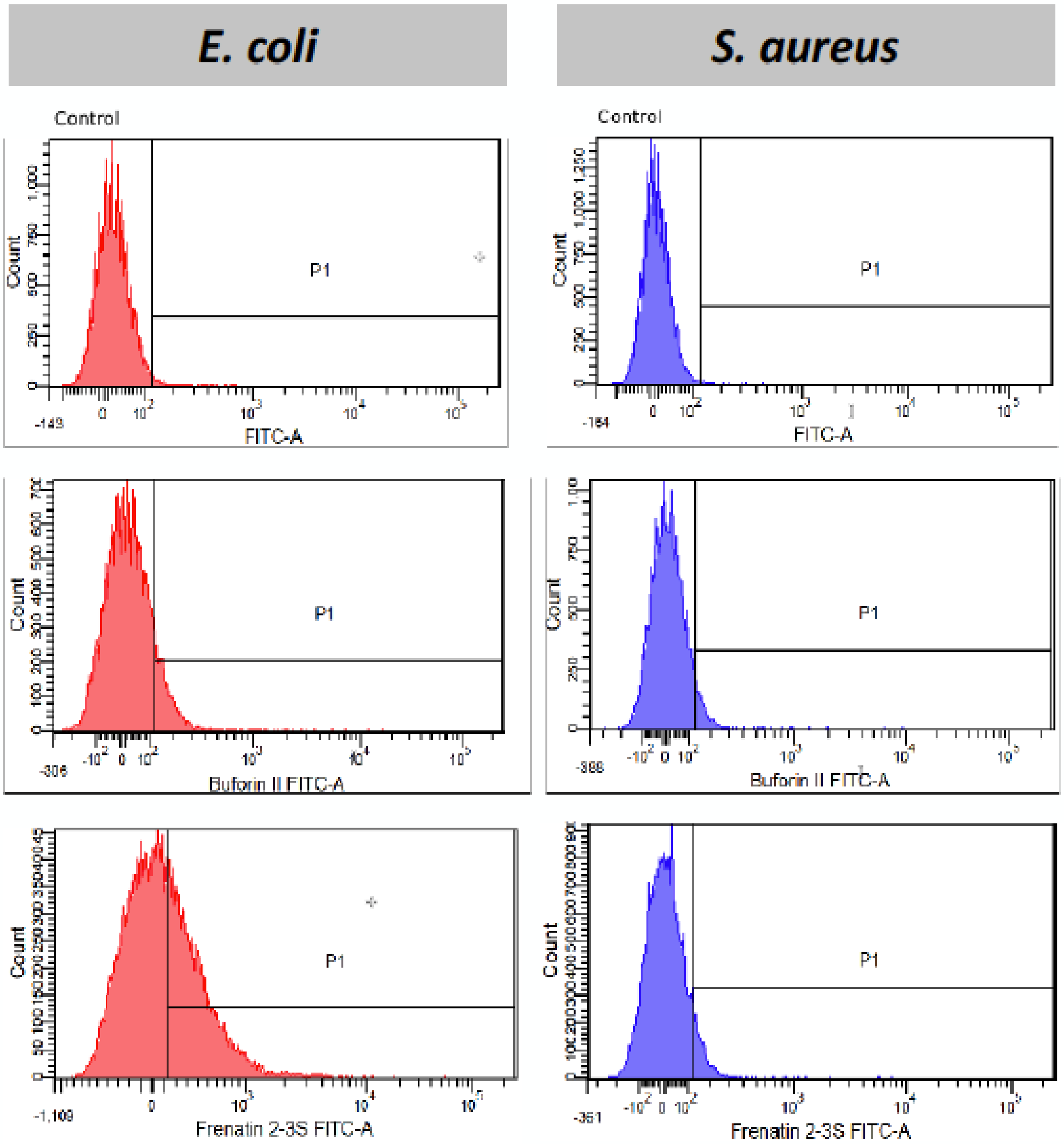

Figure S2. FACS analysis of *E. coli* and *S. aureus* cells were treated with FITC-labeled BF2 and F2.3S during 10 minutes. *E. coli* and *S. aureus* cells were grown to mid-logarithmic phase OD500= 0.2, treated with 0.5 µM of each peptide and washed with PBS and resuspended in the same buffer and analyzed by FACS.

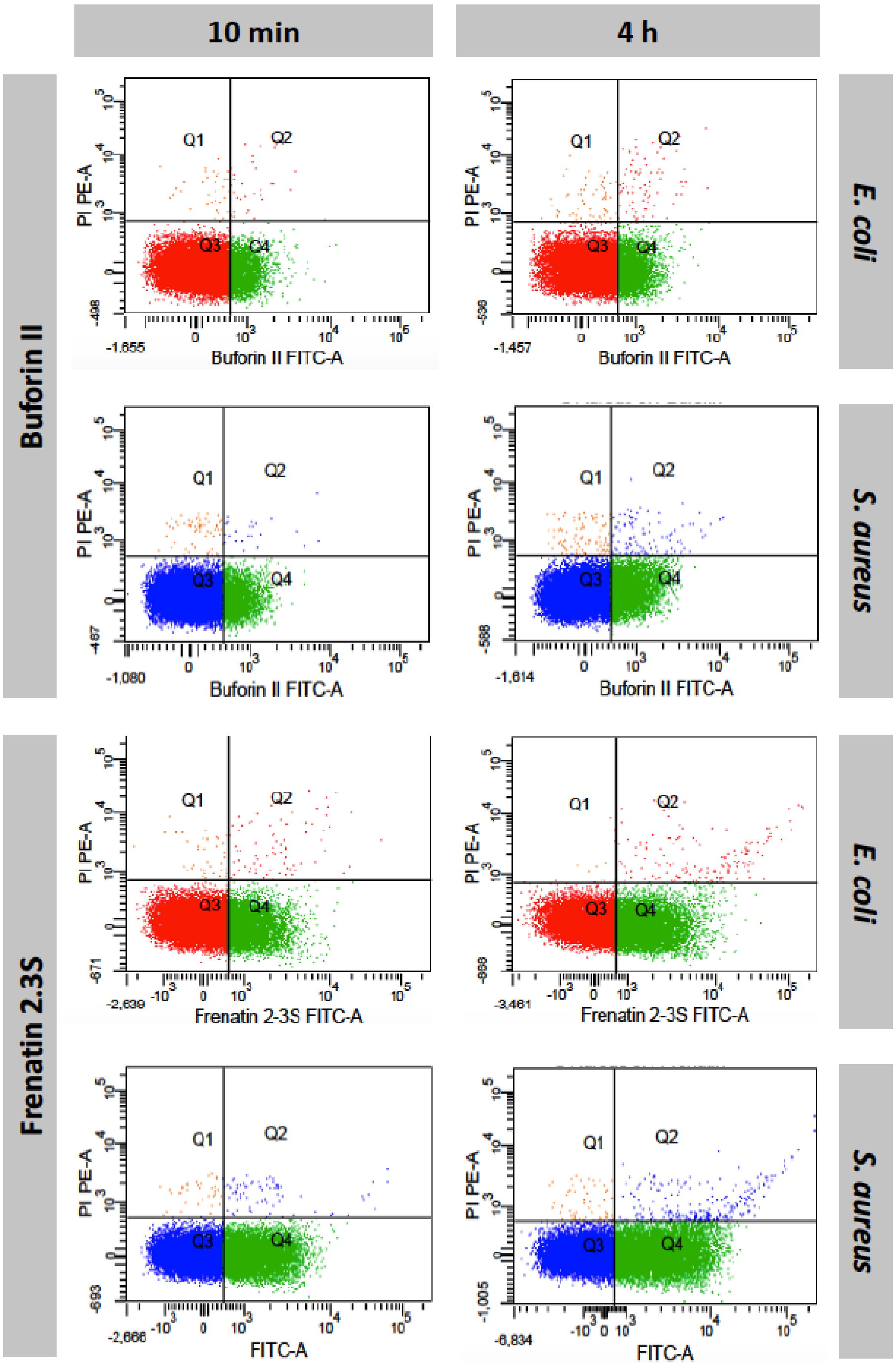

Figure S3. *E. coli* and *S. aureus* cell distribution following incubation with 0.1 µM of FITC-labelled BF2 and F2.3 peptides. Dot plot diagrams of FITC-peptide/PI at 10 min and 4 h show cell population divided in: free live bacterial cells (Q3), bacterial cells with uptake peptide (Q4), free dead bacterial cells (Q1), and dead bacterial cells with peptide uptake (Q2).

A. BF2

HPLC profile

Column: Luna C18 (4.6 × 50 mm, 3 um; Phenomenex)
Gradient: Linear B (0.036% TFA in MeCN) into A (0.045% TFA in H2O) over 15 min Flow rate: 1 mL/min
Detection: 220 nm.
Method Filename: 15a40en15.lcm

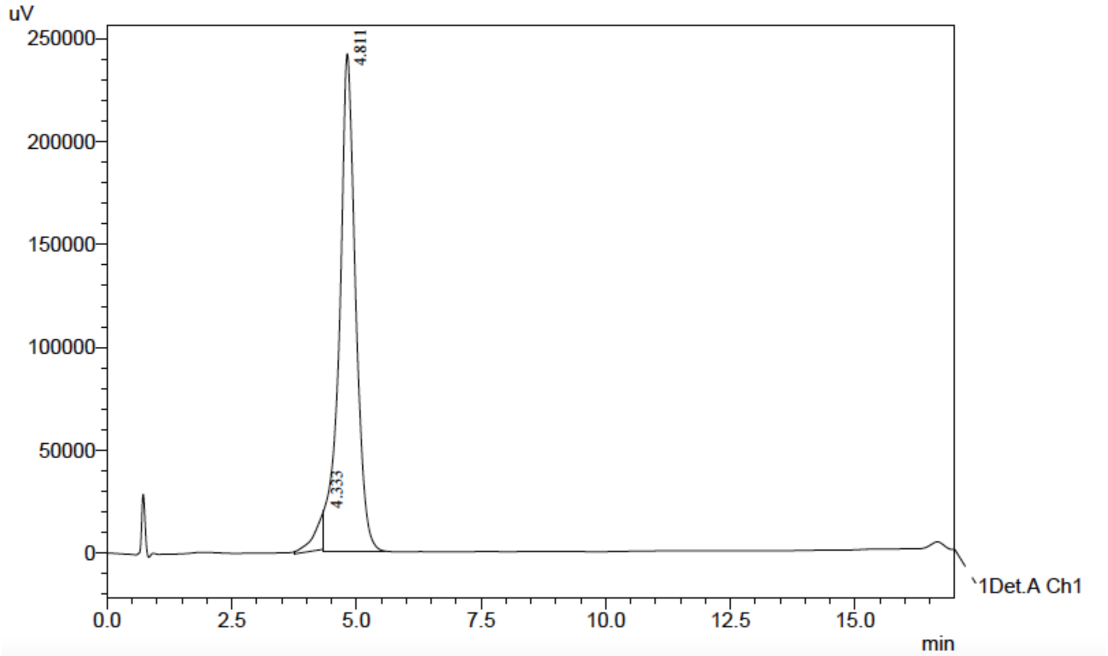

PeakTable

Detector A Ch1 220nm

Peak#	Ret. Time	Area	Height	Area %
1	4.333	238446	18171	4.169
2	4.811	5481126	242069	95.831
Total		5719573	260239	100.000

LC-MS spectra

B. LC-MS 2010EV (Shimadzu)

Column: XBridge column C18 (4.6 × 150 mm, 3.5 µm, Waters)
Gradient: A = 0.1% formic acid in water; B = 0.08% formic acid in acetonitrile
Flow rate: 15 min at a flow rate of 1 mL/min
Detection: UV detection at 220 nm

Average mass: 2434,88 Da

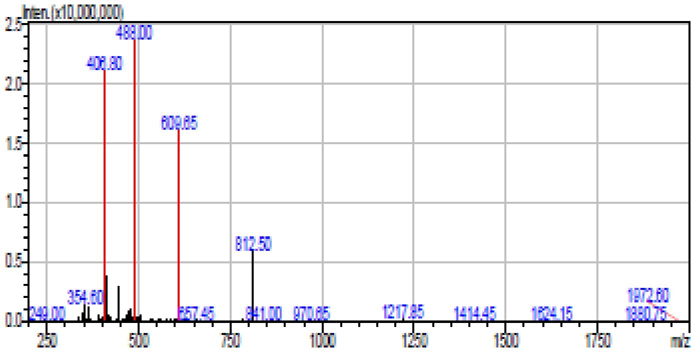

Positively charged ion series

Ch.	Average	MonoIso.
MH1+	2435.8866	2434.4378
MH2+	1218.4470	1217.7226
MH3+	812.6337	812.1508
MH4+	609.7271	609.3649
MH5+	487.9831	487.6934
MH6+	406.8205	406.5790
MH7+	348.8472	348.6402
MH8+	305.3672	305.1861
MH9+	271.5494	271.3884
MH10+	244.4952	244.3503

B. F2.3S

HPLC Profile:

Column: Luna C18 (4.6 × 50 mm, 3 um; Phenomenex)
Gradient: Linear B (0.036% TFA in MeCN) into A (0.045% TFA in H2O) over 15 min Flow rate: 1 mL/min
Detection: 220 nm.
Method Filename: 25a60en15.lcm

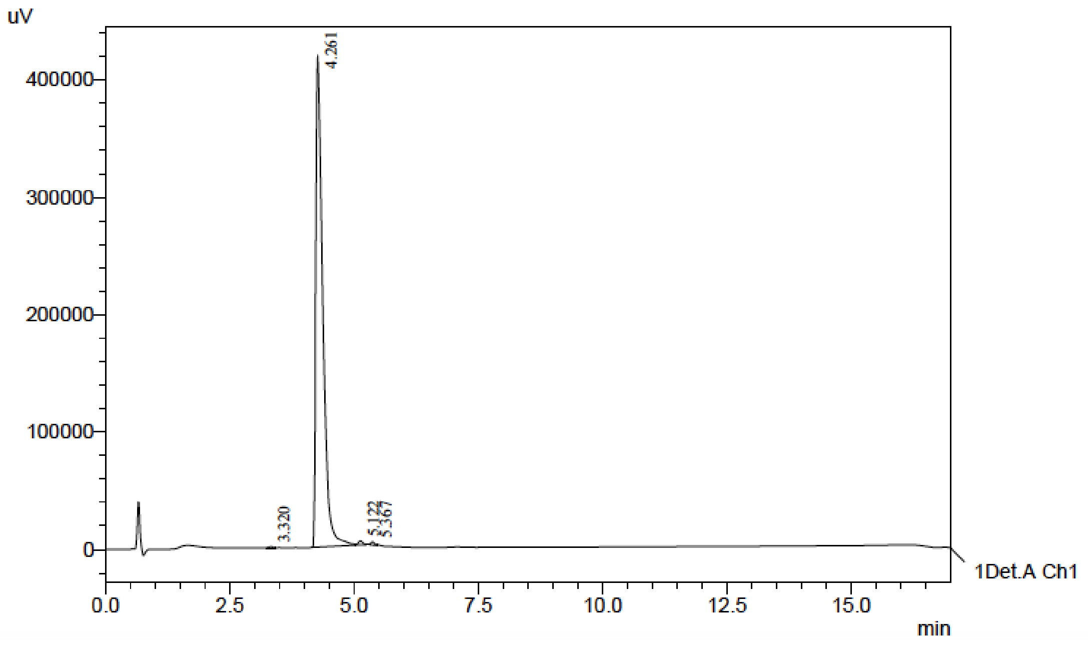

PeakTable

Detector A Ch1 220nm

Peak#	Ret. Time	Area	Height	Area %
1	3.320	16021	1952	0.373
2	4.261	4238674	419291	98.767
3	5.122	21998	3537	0.513
4	5.367	14877	2856	0.347
Total		4291570	427636	100.000

LC-MS Spectra:

B. LC-MS 2010EV (Shimadzu)

Column: XBridge column C18 (4.6 × 150 mm, 3.5 µm, Waters)
Gradient: A = 0.1% formic acid in water; B = 0.08% formic acid in acetonitrile
Flow rate: 15 min at a flow rate of 1 mL/min
Detection: UV detection at 220 nm

Average mass: 1575,92 Da

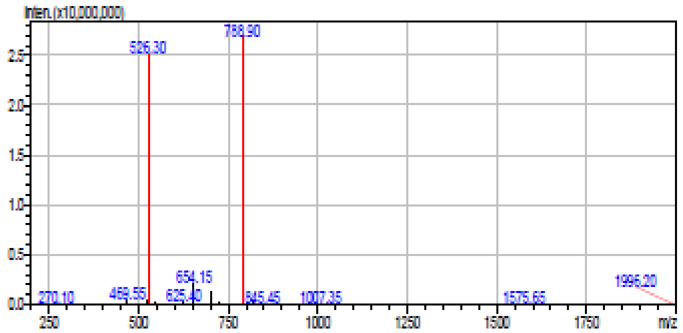

Positively charged ion series

Ch.	Average	MonoIso.
MH1+	1576.9223	1575.9581
MH2+	788.9648	788.4827
MH3+	526.3123	525.9909
MH4+	394.9860	394.7450
MH5+	316.1903	315.9974
MH6+	263.6598	263.4991
MH7+	226.1380	226.0003
MH8+	197.9967	197.8761
MH9+	176.1089	176.0018
MH10+	158.5988	158.5024

Figure S4. HPLC profiles and MS spectra of the purified peptides: A) Buforin II (BF2) TRSSRAGLQFPVGRVHRLLRK-carboxyl and B) Frenatin 2.3S (F2.3S) GLVGTLLGHIGKAILGG-carboxyl
